# Supplementary material for: NAADP‐regulated two‐pore channels drive phagocytosis through endo‐lysosomal Ca2+ nanodomains, calcineurin and dynamin
Source: EMBO J. 2020 Jun 8;39(14):e104058. doi: 10.15252/embj.2019104058 (PMC7360967; doi:10.15252/embj.2019104058)
Supplement: Supplementary file 1 — Appendix [file EMBJ-39-e104058-s001.docx]

**Appendix**

**NAADP-regulated two-pore channels drive phagocytosis through endolysosomal Ca^2+^ nanodomains, calcineurin and dynamin**

**Lianne C. Davis, Anthony J. Morgan and Antony Galione**

**Table of Contents**

**Supplementary Figures**

**Page**

**Appendix Figure S1.** Dextrans traffic to endo-lysosomes in WT BMDM…………...….…..2

**Appendix Figure S2.** BMDMs possess the NAADP-induced Ca^2+^-release pathway....…..3

**Appendix Figure S3.** TPCs localize to acidic organelles………….…………………...…….4

**Appendix Figure S4.** FcγR activation induces peri-lysosomal (TPC-mediated) Ca^2+^ signals, but not peri-lysosomal pH changes…………...……………..5

**Appendix Figure S5.** FcR stimulation evokes Ca^2+^ nanodomains across the lysosomal network………………………………….………………………………..6

**Appendix Figure S6.** Cytosolic Ca^2+^ and calcineurin activity monitored simultaneously

in single macrophages………………………….………………………7

**Appendix Figure S7.** Western blot detection of phospho-dynamin-2 reveals that phagocytosis induces dynamin dephosphorylation is TPC1-dependent……………………………………….……………………….8

**Supplementary Figures**

**
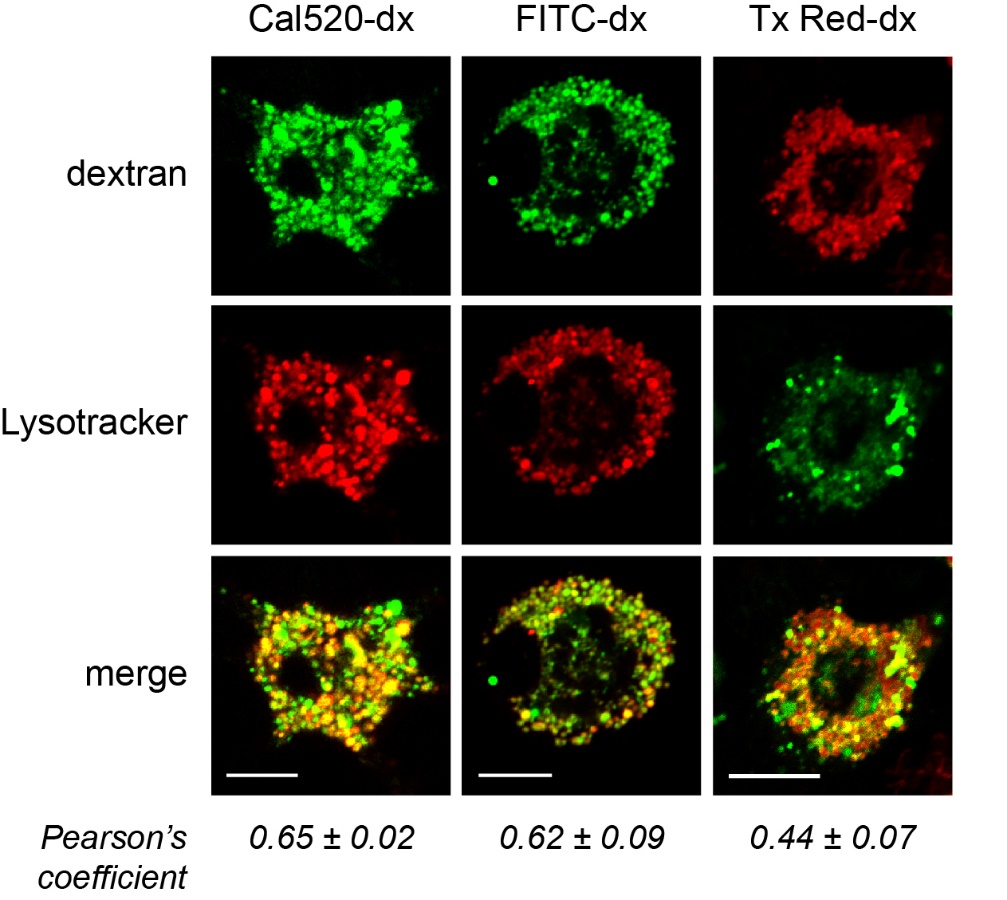
**

**Appendix Figure S1. Dextrans traffic to endo-lysosomes in WT BMDM.**

Related to Figure 1.

Ca^2+^-binding-dextran (Cal 520) and non-Ca^2+^-binding-dextrans (FITC and Texas Red) were endocytosed by WT BMBM and trafficked to the endo-lysosomes, as indicated by co-labelling with an orthogonal Lysotracker. Co-localization is shown in yellow and quantified using Pearson’s correlation coefficient. All scale bars = 10 μm.

**
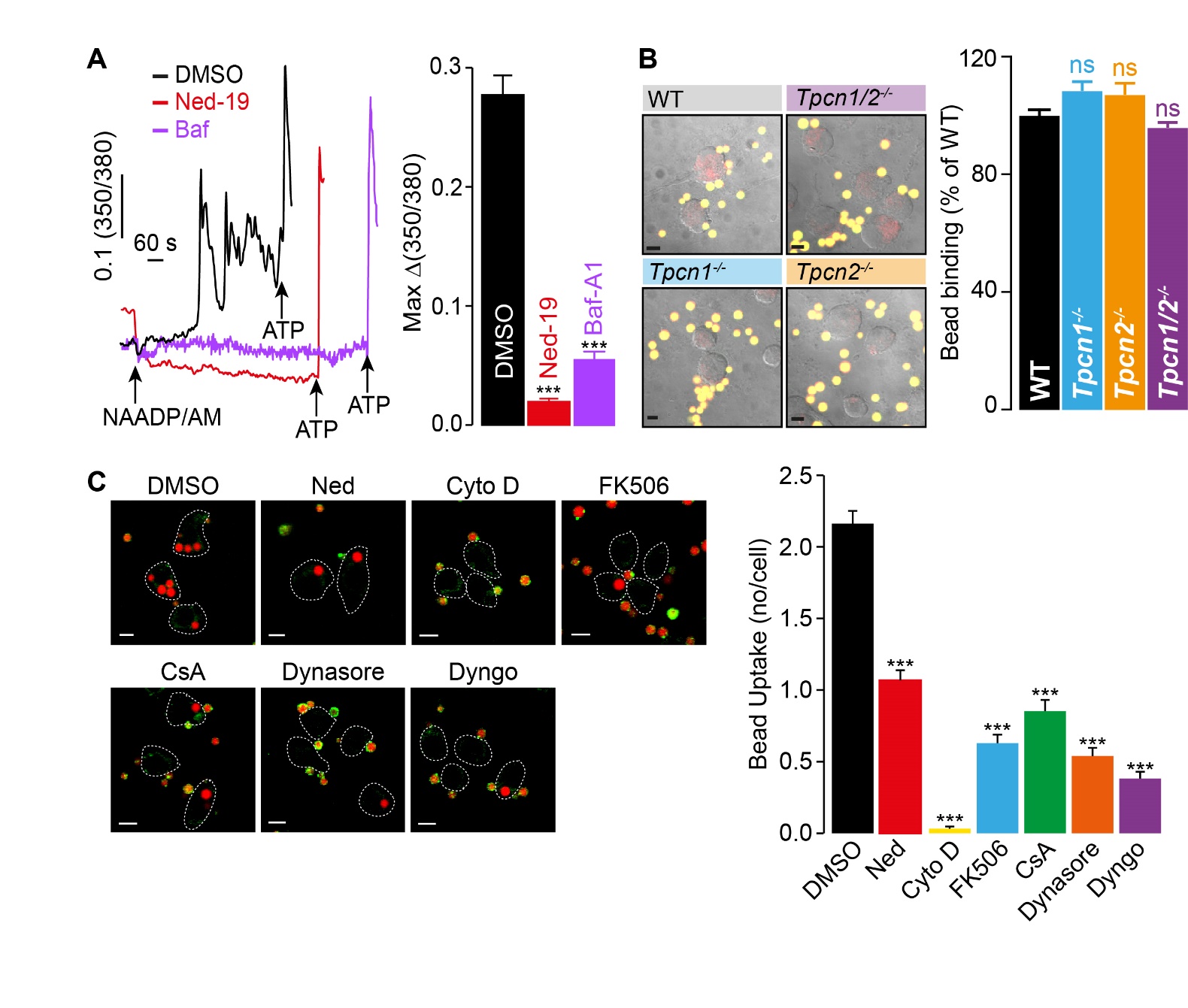
**

**Appendix Figure S2. BMDMs possess the NAADP-induced Ca^2+^-release pathway.**

Related to Figure 2.

(A) Cytosolic Ca^2+^ signals with 10 μM extracellular NAADP/AM in WT BMDM were inhibited by Ned-19 (10 μM) or by depletion of acidic Ca^2+^ stores with Bafilomycin A1 (1 μM). Representative single-cell traces show 350/380 ratios of fura-2 fluorescence and the subsequent maximum response to 100 μM ATP. Collated maximum peak 350/380 ratio changes to NAADP/AM; mean ± SEM, n = 339 (DMSO), 158 (Ned-19), 85 (Baf-A1) cells. (B) IgG-bead binding is not inhibited in BMDMs from *Tpcn^-/-^* mice. Representative images of BMDM treated with 10 μM cytochalasin D to prevent IgG-3 μm bead internalization (scale bars = 5 μm), but not binding to the cell surface (FcγR); expressed as a percentage of WT in population assays. n = 28 (WT), 16 (*Tpcn1^-/-^*), 16 (*Tpcn2^-/-^*), 12 (*Tpcn1/2^-/-^*) wells of 96-well plate. Non-significant P=0.0665 (one-way ANOVA). (C) Phagocytosis of IgG-3-μm beads by RAW 246.7 macrophages, was inhibited by Ned-19 (10 μM) n=235 cells, cytochalasin D (10 μM) n=128 cells, FK506 (10 μM) n=194 cells, CsA (10 μM) n=156 cells, dynasore (50 μM) n=224 cells and Dyngo-4a (10 μM) n=194 cells, compared to control DMSO (0.1%) n=198 cells. Representative images of single cells, delineated by dotted lines, 30 min after phagocytosis; internalized beads in red, external beads in green/yellow (scale bars = 5 μm). Mean number of internalized beads per cell ± SEM. ***P<0.001, **P<0.01 (one-way ANOVA).

**
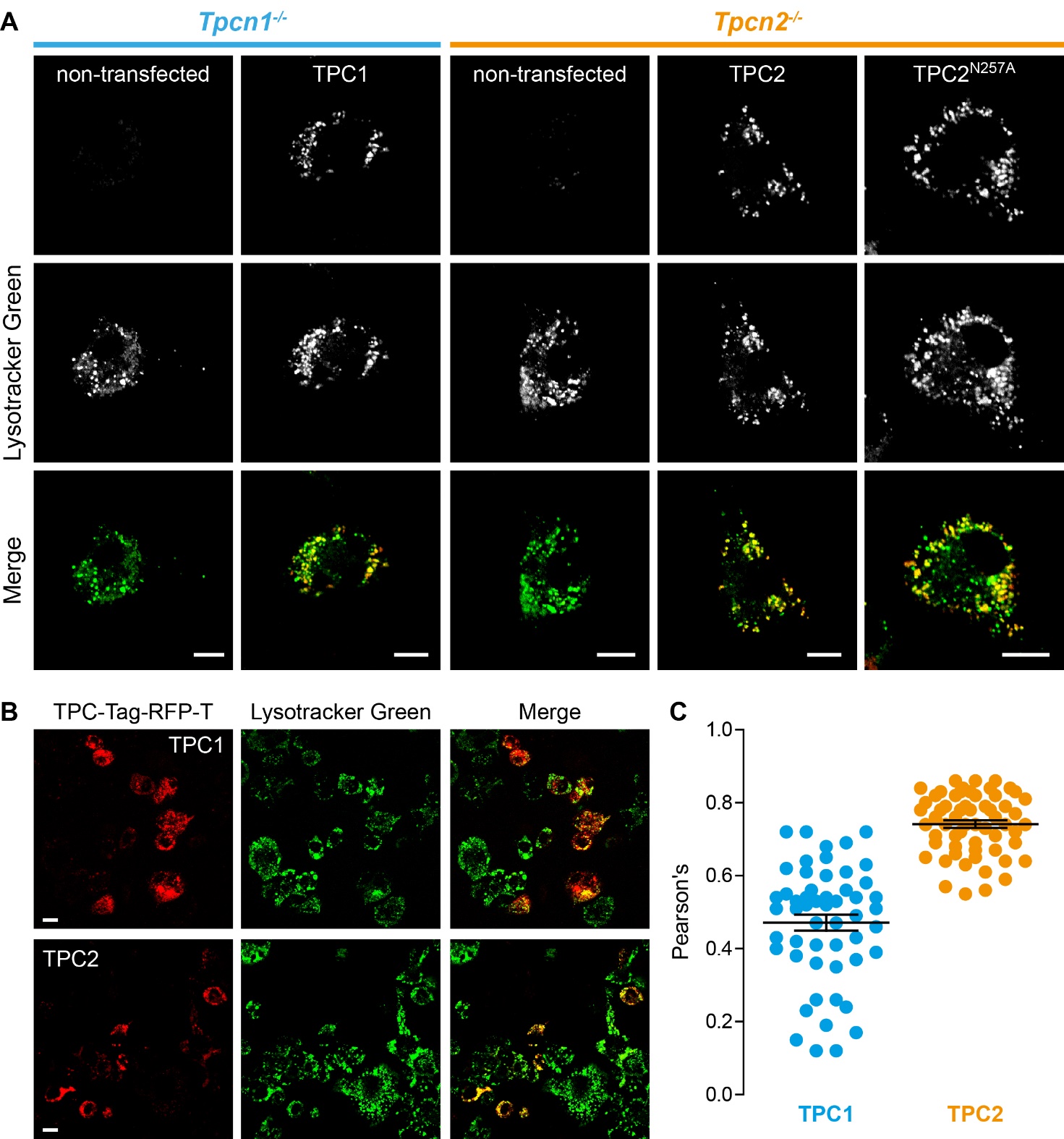
**

**Appendix Figure S3. TPCs localize to acidic organelles.**

Related to Figure 2.

(A) BMDMs from *Tpcn1^-/-^* or *Tpcn2^-/-^* mice heterologously-expressing mouse TPC versions tagged with Tag-RFP-T (in red in the merge image) were labelled with Lysotracker Green (in green in the merge image). BMDM not-transfected with TPC constructs (non-transfected) did not show any fluorescence signal in the red channel. Co-localisation of TPC and Lysotracker (in yellow) is indicative of an acidic organelle localization and was assessed by Pearson’s coefficient: TPC1 0.79 ± 0.022 (n = 8 cells); TPC2 0.80 ± 0.012 (n = 23 cells); TPC2 (N257A) 0.66 ± 0.033 (n = 13 cells). (B-C) RAW cells heterologously-expressing mouse TPC versions tagged with Tag-RFP-T were labelled with Lysotracker Green and co-localization assessed by plotting the Pearson’s coefficient of each single cell with the mean ± S.E.M. in black: TPC1 0.47 ± 0.022 (n = 53 cells); TPC2 0.74 ± 0.011 (n = 60 cells). All scale bars = 10 μm.

**
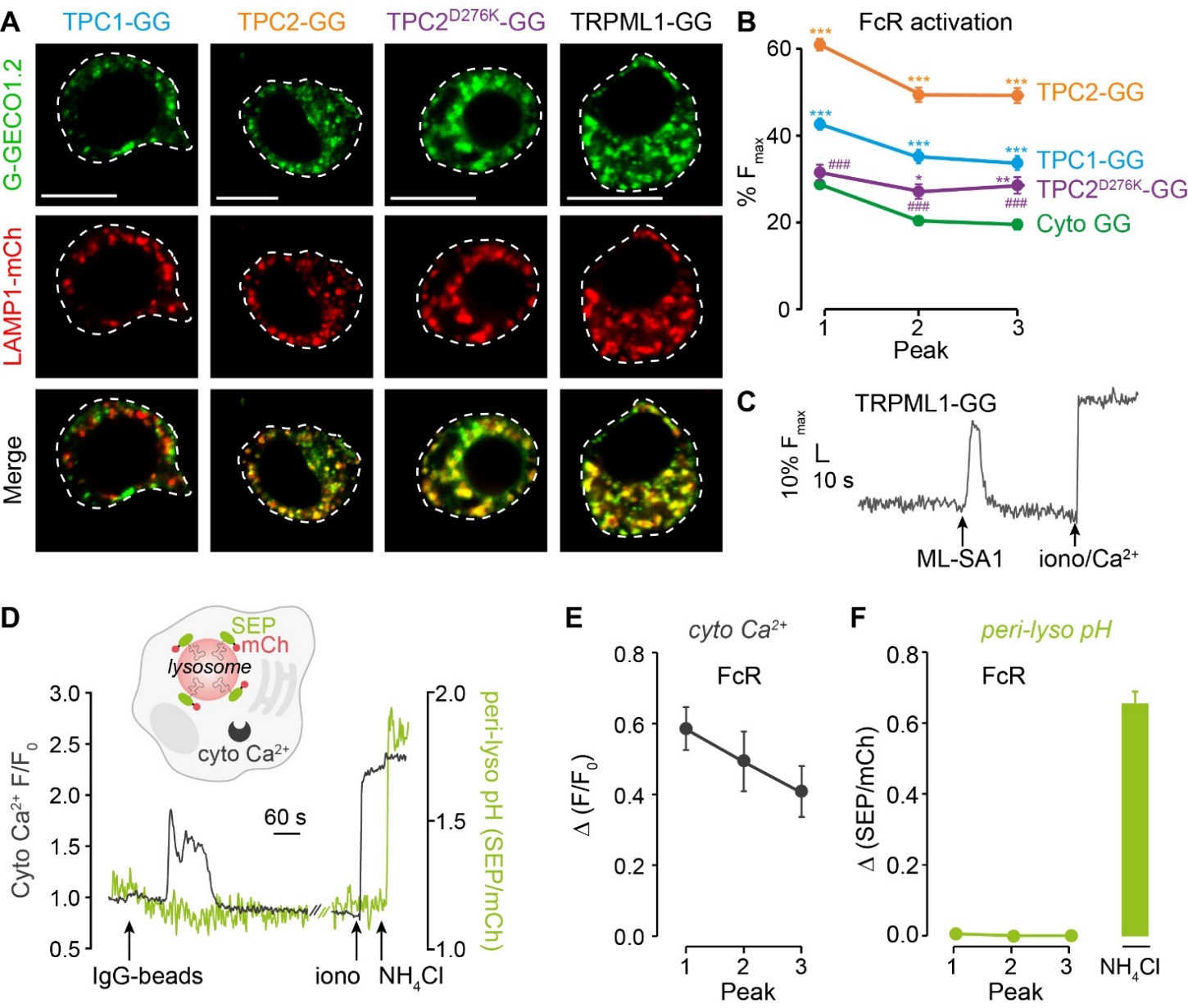
Appendix Figure S4. FcγR activation induces peri-lysosomal (TPC-mediated) Ca^2+^ signals, but not peri-lysosomal pH changes.**

Related to Figure 5 and 6.

(A) RAW 247.6 macrophages expressing TPC- or TRPML1-tethered G-GECO1.2 were imaged immediately upon addition of 1 μM ionomycin and 5 mM CaCl_2_ since the basal fluorescence of G-GECO1.2 was not sufficiently bright for co-localization analysis. TPC2-G-GECO1.2, the TPC2 D276K mutant (that blocks ion permeation) and TRPML1-G-GECO1.2 are localised to lysosomes, as indicated by their significant co-localization with LAMP1-mCherry: yellow puncta in the merged image, and Pearson’s coefficients of 0.91 ± 0.015, 0.83 ± 0.011 and 0.82 ± 0.011, respectively. TPC1-G-GECO1.2 is only partially localised to lysosomes, Pearson’s coefficient of 0.43 ± 0.03. All scale bars = 10 μm. (B) Summary data of the first three Ca^2+^ peaks (G-GECO1.2 signal) evoked by dropping IgG-3-μm beads onto RAW 247.6 expressing TPC-tethered G-GECO1.2 or cytosolic G-GECO1.2 (cyto GG) in –Ca^2+^_o_ ECM, n = 76-274 cells. ***P<0.001, **P<0.01, *P<0.05 vs cyto GG; ###P<0.001 TPC2-GG vs TPC2(D276K)-GG (one-way ANOVA). (C) Single-RAW cell trace showing responsivity of TRPML1-G-GECO1.2 to the TRPML agonist, ML-SA1 (50 μM). (D) Simultaneous recording of cytosolic Ca^2+^ and peri-lysosomal pH following FcγR-activation by IgG-3-μm beads in –Ca^2+^_o_ ECM. Cytosolic Ca^2+^ oscillations (detected using cytosolic B-GECO1) were evoked (E), whilst no changes in peri-lysosomal pH were detected ratiometrically (using LAMP1-SEpHluorin-mCherry) in the same single RAW 246.7 macrophage (F). As a positive control, addition of NH_4_Cl (10 mM) to alkalinize the cytosol confirmed that the peri-lysosomal-targeted SEpHluorin (SEP) was responsive to changes in pH (D, F), n = 20 cells.


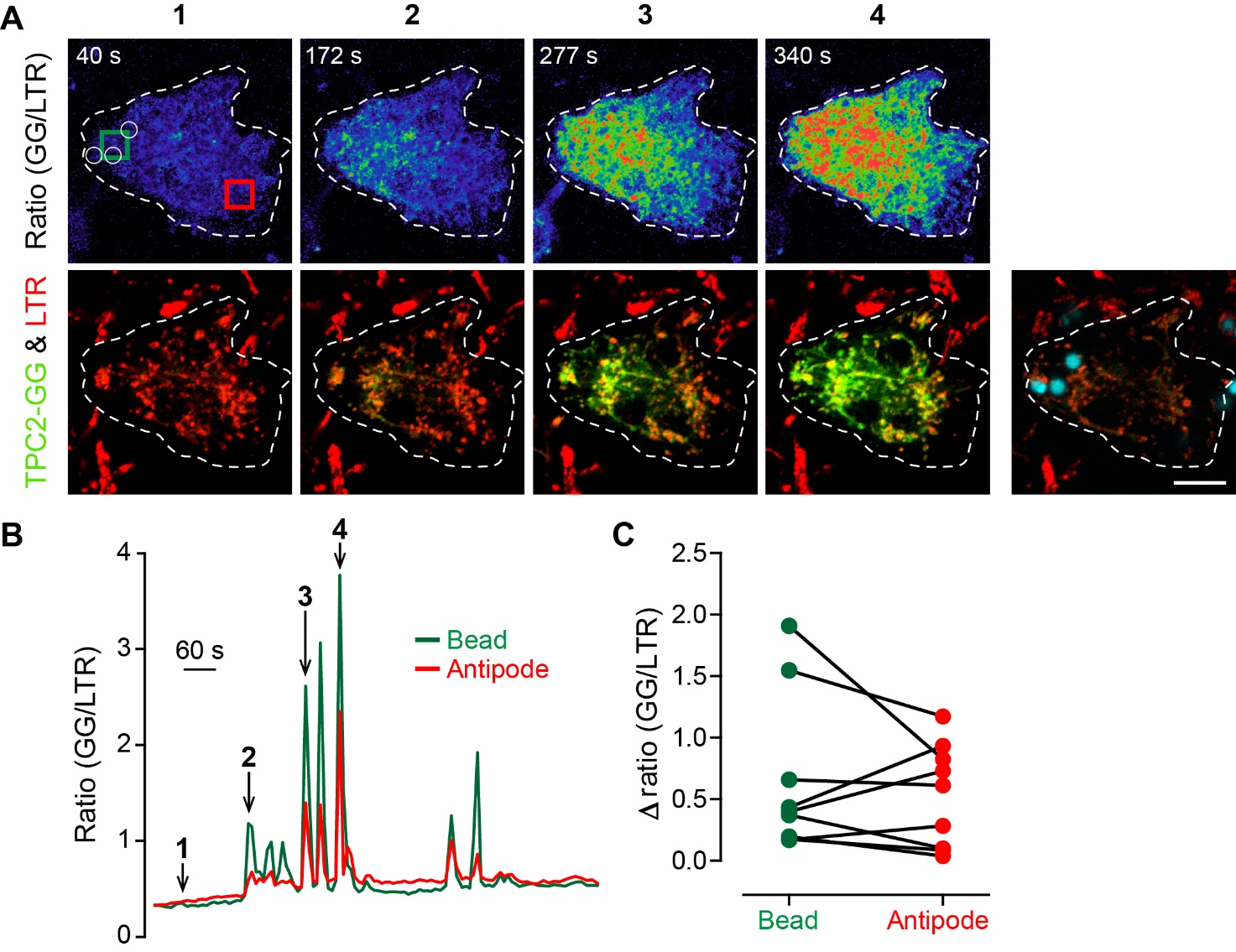


**Appendix Figure S5. FcR stimulation evokes Ca^2+^ nanodomains across the lysosomal network.**

Related to Figure 5.

RAW246.7 cells transfected with TPC2-G‑GECO1.2 were loaded with 200 nM Lysotracker Red (LTR) for 5 mins and presented with 3-µm opsonized beads labelled with Alexa Fluor 647 (blue) with images collected every 7-10 s. The sites of bead engagement are overlaid as white circles (taken from the end micrograph blue beads). (A) At the indicated times, upper pseudo-coloured images of the G‑GECO1.2/LTR ratio, and lower equivalent overlays of the TPC2-G‑GECO1.2 (green) and LTR (red) channels. (B) Ca^2+^ signals plotted as the TPC2-G‑GECO1.2/LTR ratio at the bead engagement site (green square) and the cell antipode (red square). Numbered arrows indicate the times of the images in (A). (C) Paired Bead and Antipode values for individual cells, where each point is the mean Ca^2+^ peak of multiple spikes (1-5 spikes from 9 cells). Although the single cell in A,B shows a difference between bead and antipode, lysosomes were still activated distal to the the engagement site and, overall, there was no significant difference between the bead and antipode amplitudes (P>0.2, paired *t* test). Scale bar = 10 µm.

**Appendix
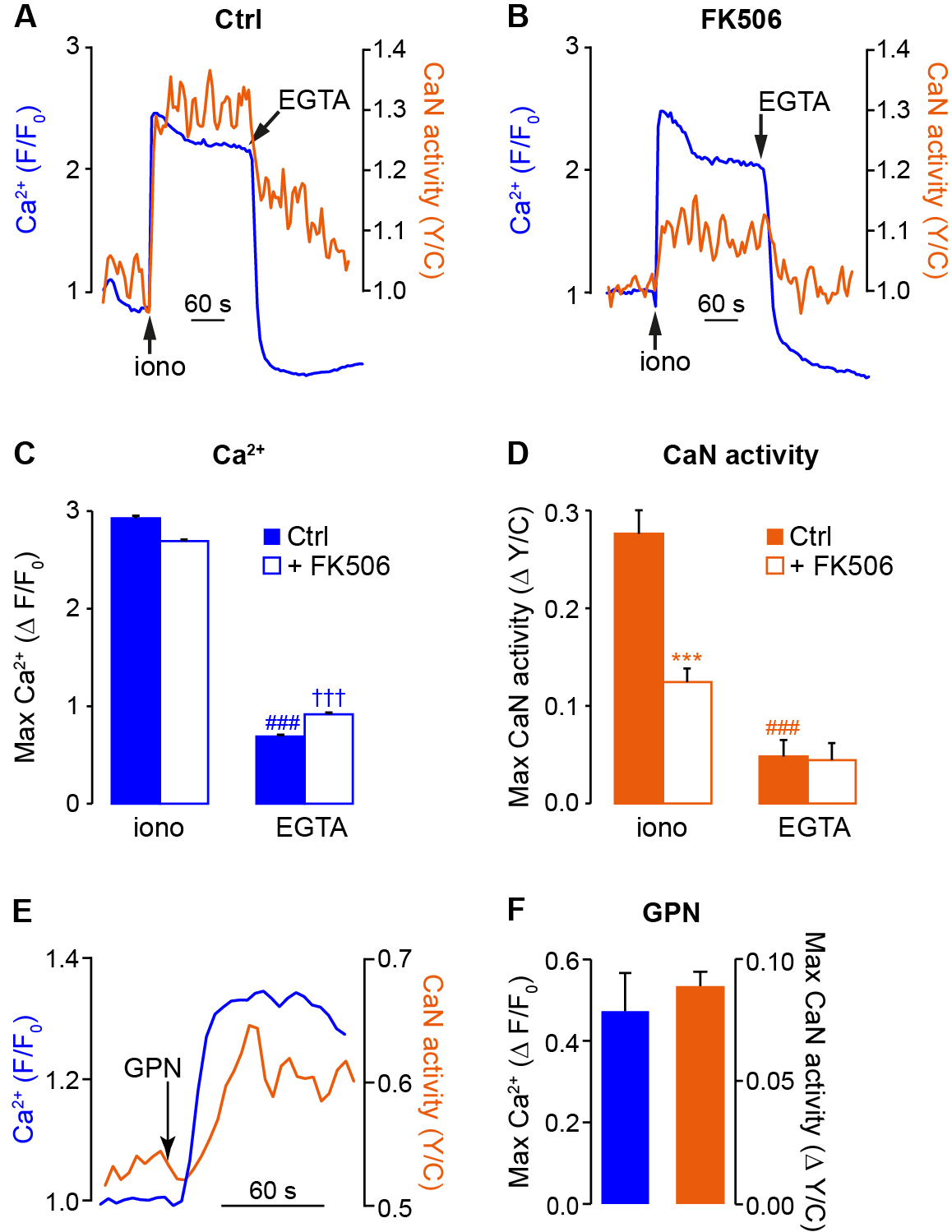
Figure S6. Cytosolic Ca^2+^ and calcineurin activity monitored simultaneously in single macrophages.**

Related to Figure 7.

In RAW 264.7 macrophages, cytosolic Ca^2+^ responses (in blue) and cytosolic calcineurin (CaN) activity (in orange) were simultaneously monitored using genetically encoded reporters, jRGECO1a and the FRET biosensor CaNAR2, respectively. (A, B, E) Representative time-course of single cells showing fluorescence Ca^2+^ changes normalized to initial fluorescence (F/F_0_) and YPet/Cerulean3 (Y/C) emission ratio changes (FRET) from CaNAR2 (a 5^th^ order Savitzky-Golay smoothing filter has been applied). (A-D) Cells were stimulated with 2 μM ionomycin with (“+FK506”, n = 9) or without (“Ctrl”, n = 27) 10 μM of the calcineurin inhibitor FK506, followed by addition of 5 mM EGTA. ***P<0.001: Ctrl vs +FK506, ### P<0.001: Ctrl iono vs ctrl EGTA, ††† P<0.001: +FK506 iono vs +FK506 EGTA. (E, F) Cells were stimulated with 200 μM GPN (n = 20 cells). Graphs presented as the mean ± S.E.M. and probability determined using one-way ANOVA.

**
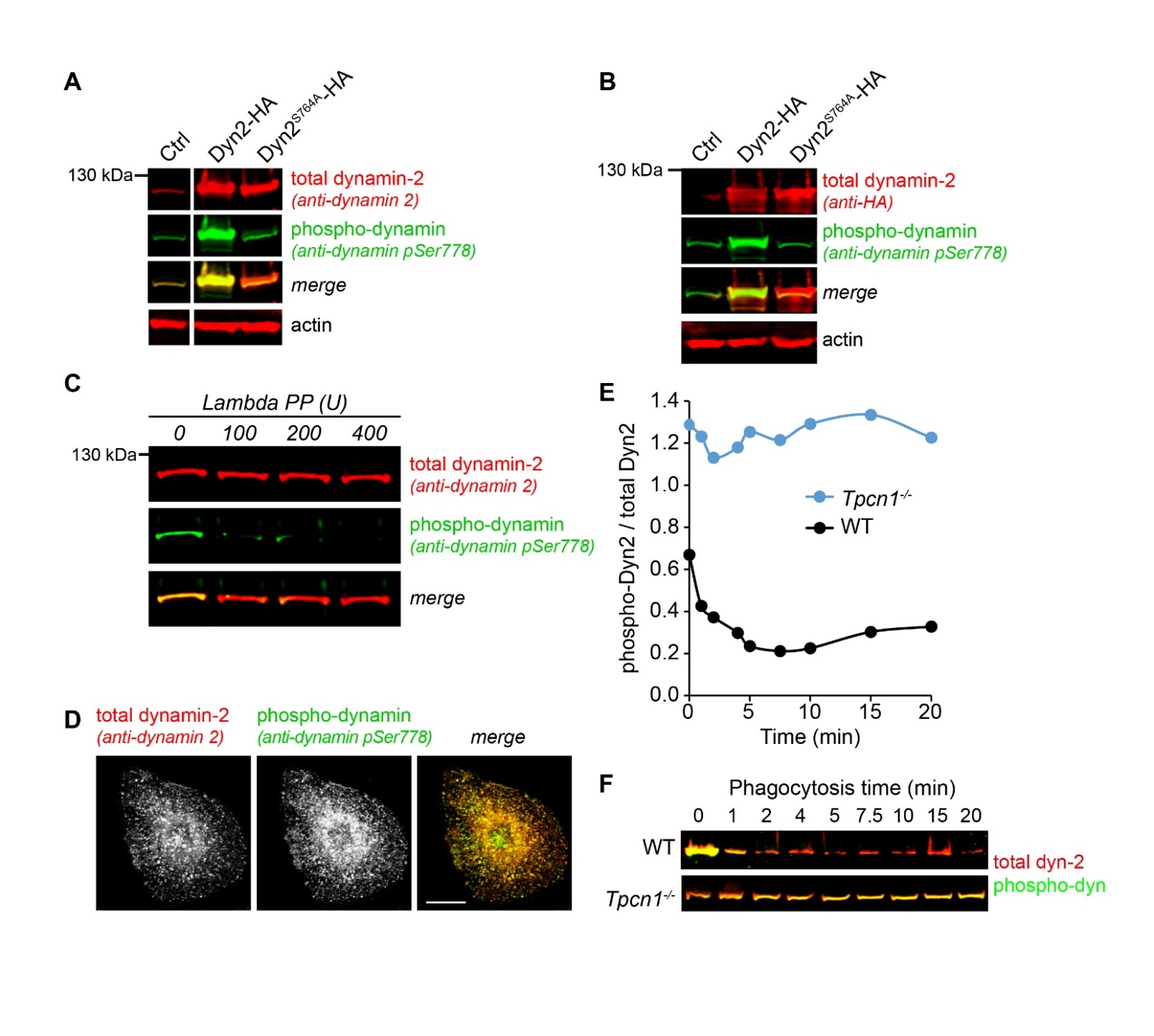
Appendix Figure S7. Western blot detection of phospho-dynamin-2 reveals that phagocytosis induces dynamin dephosphorylation is TPC1-dependent.**

Related to Figure 7.

Two-colour immunoblot detection of total dynamin-2 (~95 kDa, in red) probed with (A) anti-dynamin-2 or (B) anti-HA, as well as phosphorylated dynamin-2 (in green) probed with anti-dynamin pSer778. Non-transfected COS-7 cells (Ctrl) show immunolabelling of endogenous dynamin-2 (total and phosphorylated). Immunoreactivity of anti-dynamin pSer778 was increased in COS-7 cells transiently expressing wildtype dynamin-2 (Dyn2-HA); this increase was prevented by expressing the dynamin-2 S764A mutant (Dyn2^S764A^-HA), which cannot be phosphorylated. HA denotes the hemagglutinin epitope tag. (C) Analysis of total WT BMDM lysate showing immunolabelling of total dynamin-2 and phospho-dynamin. The phospho-specificity of this immunolabelling is shown by lambda protein phosphatase (Lambda PP) treatment, which removes phosphate groups from phosphorylated residues (serine 764) in dynamin-2. (D) Immunocytochemistry of total dynamin-2 and phospho-dynamin in WT BMDM confirmed their co-localization. Scale bar = 10 μm. (E, F) Analysis of dynamin phosphorylation in BMDM from WT and *Tpcn1^-/-^* mice during phagocytosis of rabbit-IgG 3-μm beads. The intensity of the phospho-dynamin immunoreactive band on western blot (in green (F)) was normalised to the intensity of the total dynamin immunoreactive band (in red (F)) and plotted against the time course of the phagocytosis assay (E). FcR-mediated phagocytosis induced a dephosphorylation of dynamin, which was abolished in the TPC1 knockout (this agrees with the data shown in Fig. 6J using the in-cell western assay).
