# Supplementary material for: NAADP‐regulated two‐pore channels drive phagocytosis through endo‐lysosomal Ca2+ nanodomains, calcineurin and dynamin
Source: EMBO J. 2020 Jun 8;39(14):e104058. doi: 10.15252/embj.2019104058 (PMC7360967; doi:10.15252/embj.2019104058)

Source Data: Appendix Fig. S7A

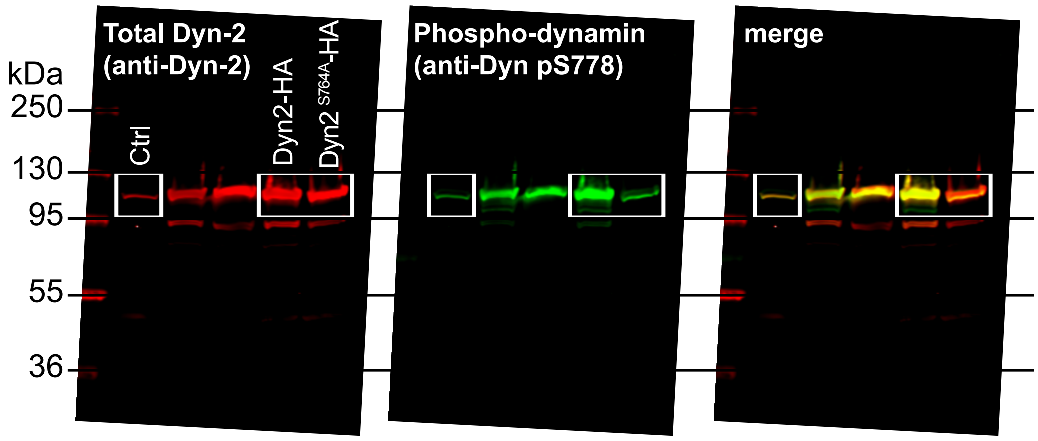

Source Data: Appendix Fig. S7B

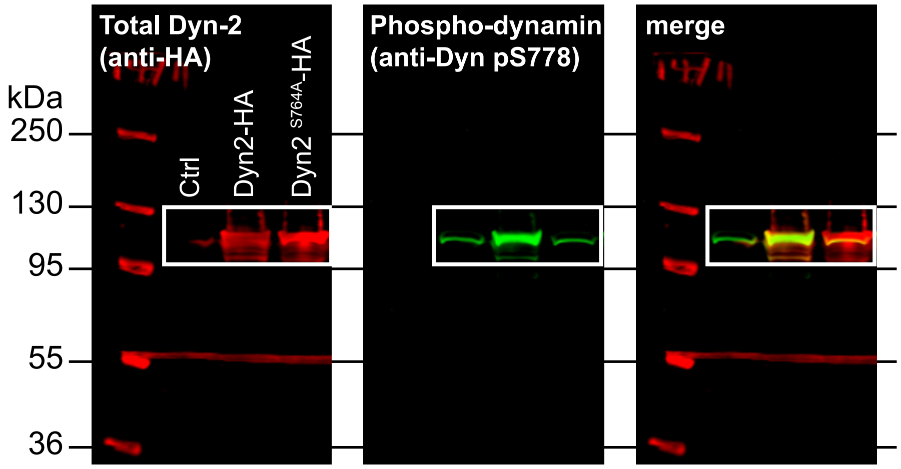

Source Data: Appendix Fig. S7C

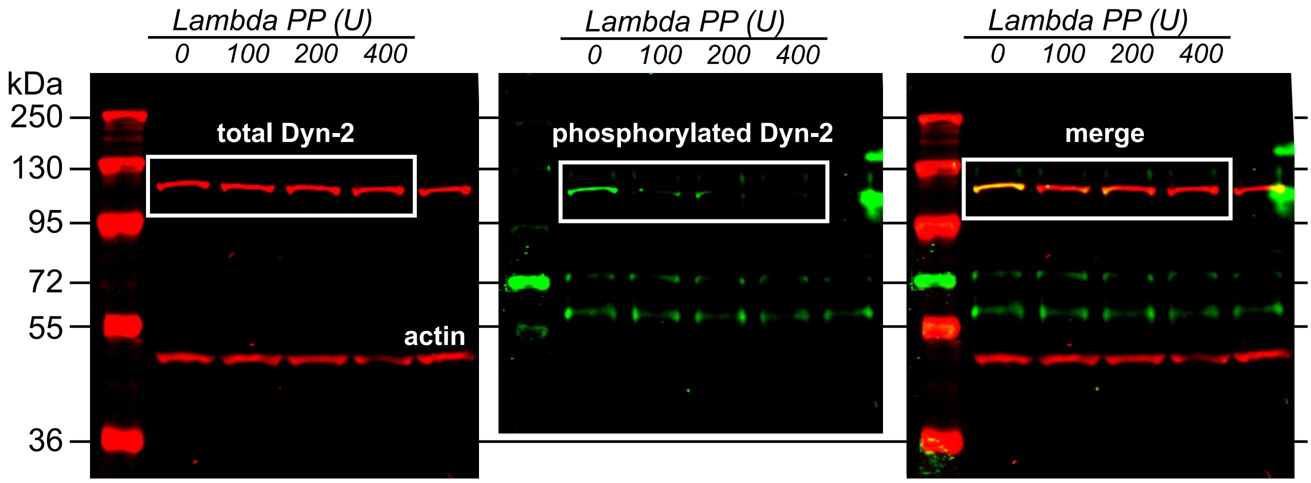

Source Data: Appendix Fig. S7F

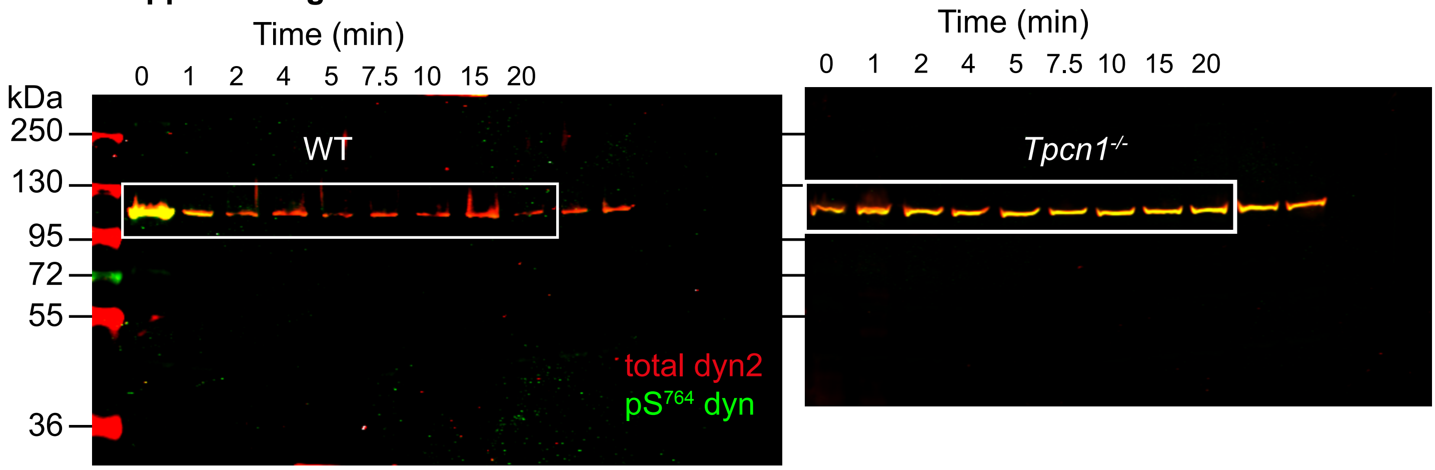

Supplement: Supplementary file 2 — Source Data for Appendix [file EMBJ-39-e104058-s002.zip › EMBOJ-2019-104058R1-Appendix_Figure_S7_Source_Data-sd.pdf]
